# ADHD Children Take More Time to Inhibit Automatic Responses: A Comparison with Anxiety Disorders Using NEPSY-II

**DOI:** 10.3390/children12060798

**Published:** 2025-06-18

**Authors:** Fabiola Panvino, Valerio Zaccaria, Michela Pica, Nunzia Amitrano, Francesco Pisani, Carlo Di Brina

**Affiliations:** Unit of Child and Adolescent Neuropsychiatry, Department of Human Neuroscience, Sapienza University of Rome, Via dei Sabelli 108, 00185 Rome, Italy; valerio.zaccaria@uniroma1.it (V.Z.); m.pica3@student.unisi.it (M.P.); nunzia.amitrano@uniroma1.it (N.A.); francesco.pisani@uniroma1.it (F.P.); c.dibrina@policlinicoumberto1.it (C.D.B.)

**Keywords:** ADHD, anxiety disorders, inhibition, attention, working memory, neuropsychology

## Abstract

Background/Objectives: Attention deficit hyperactivity disorder (ADHD) and anxiety disorders (AD) are prevalent in childhood and adolescence, often presenting with overlapping symptoms. This study aimed to assess cognitive and executive functions—specifically attention, inhibition, and working memory—in children with ADHD, AD, and comorbid ADHD + AD. It also sought to identify potential neuropsychological markers that differentiate ADHD from AD and ADHD + AD comorbidity. Methods: Cognitive and executive functions were assessed in a sample of 48 school-age children and adolescents (aged 8–15 years) using the Wechsler Intelligence Scale for Children—Fourth Edition (WISC-IV) and the NEPSY-II battery. The MASC 2 self-report questionnaire was also used to assess anxiety symptoms. The participants were divided into three groups based on clinical diagnoses: ADHD, AD, and ADHD + AD. Results: No significant group differences emerged in cognitive performance, attention, or working memory. Significant differences emerged in inhibition performance, with children in the AD group demonstrating more efficient inhibition compared to both the ADHD and ADHD + AD groups. Children with ADHD showed longer response times. Better performance on inhibition tasks was associated with more severe anxiety symptoms. Conclusions: These findings suggest that anxiety may modulate specific aspects of executive functioning during tasks requiring attentional and inhibitory control. However, the complex interaction between ADHD and anxiety requires further investigation. This study underscores the importance of distinguishing ADHD from AD based on cognitive and executive profiles, particularly inhibition. In this context, it supports the routine use of the NEPSY-II in combination with the MASC 2 questionnaire to facilitate differential diagnosis in clinical practice.

## 1. Introduction

Attention span deficits and distractibility are commonly reported by teachers and parents of school-age children, often prompting families to seek consultation with child neuropsychiatry services. These symptoms, although commonly seen in academic settings, are not limited to school-related tasks and may be linked to underlying conditions, such as attention deficit hyperactivity disorder (ADHD) and anxiety disorders (AD) [1,2,3,4,5].

ADHD is a common neurodevelopmental disorder, affecting approximately 5% of children worldwide, with a male predominance (3:1 ratio) before age 12 years, which becomes more pronounced during adolescence (6:1) [6]. Its core symptoms are hyperactivity–impulsivity and inattention; while the former are typically of an earlier age and tend to decrease with growth, inattention remains stable and predominant during adolescence and adulthood [7,8,9,10,11,12,13]. Hyperactivity/impulsivity symptoms are defined by restlessness and fidgetiness during any calm activities, disinhibition and interruptions, and aggressive behavior [14]. These behaviors may be perceived as intentional and bothering by parents, teachers, and peers [15], turning into social rejection. Inattention is characterized by distractibility, carelessness, frequent daydreaming, and forgetfulness; its consequences on academic performance and social interaction are more evident in adolescence as school and social expectations increase [16].

Both subtypes are associated with social impairments and emotional dysregulation, which tend to persist across the lifespan [17,18]. These difficulties are likely linked to cognitive impairments in both verbal and performance domains along with deficits in executive functions (EF), such as working memory, cognitive flexibility, and inhibitory control—key processes involved in emotion and behavior regulation [19,20].

Diagnosing ADHD can be challenging due to the absence of specific biomarkers and the overlap of ADHD symptoms with other disorders [21]. Comorbidities with various internalizing and externalizing disorders are common and complicate the diagnostic process [22]. ADHD shares several features of inattention with specific learning disorders (SLDs), AD, and depressive disorders, making differential diagnosis difficult, especially in youth [7]. Children with SLDs may develop secondary anxious symptoms due to academic struggles [23], while those with AD may show concentration difficulties due to pervasive worries, hypervigilance, and rumination. AD are defined by excessive and enduring fear, worry and apprehension, and/or the avoidance of perceived threats either in the external (for example, social situations) or internal (for example, bodily sensations) environment. They typically start early in life and include several subtypes, such as generalized, social, and separation anxiety disorders [7].

Interestingly, while several theoretical models conceptualize cognitive and EF deficits as core mechanisms underlying ADHD [24,25,26,27,28], in anxiety disorders, cognitive and EF impairments are generally considered either outcomes of the disorder or mechanisms contributing to its development and maintenance [4,18,29,30]. Higher levels of trait anxiety in childhood and adolescence have been associated with deficits in working memory and inhibitory control in some studies, although the findings are mixed [31,32,33,34,35]. This variability may reflect differences in measurement methods, anxiety conceptualization, or lack of control for comorbid ADHD symptoms [36].

In the past, ADHD and AD were considered mutually exclusive [37]. However, more recent epidemiological and clinical studies have highlighted a close relationship between ADHD and AD. It should be noted that AD are the most frequent comorbidities observed in children and adolescents with ADHD, as research suggests that up to 50% of children with ADHD also experience anxiety symptoms [38,39].

ADHD has been proposed as a potential risk factor for later development of AD [40], especially for school children, adolescents, and young adults, who often show a reduction in hyperactivity and a persistence of inattention symptoms [12,22,37,41,42]. Attention deficits and poor academic performance may negatively impact peer relationships, family dynamics, and self-esteem, thereby contributing to the secondary emergence of AD [37,43,44]. Conversely, AD may exacerbate inattention and working memory deficits in children with ADHD while partially attenuating impulsivity and response inhibition difficulties [45,46,47]. This bidirectional influence underscores the complexity of the comorbid presentation, which may vary depending on developmental stage and symptom expression.

Interestingly, some authors have proposed that, in early development, hyperactivity/impulsivity symptoms may offer a modest protective effect against the emergence of emotional problems or may mask them, contributing to their under-detection [43,48,49]. However, ADHD and AD comorbidity has also been documented in pre-school children with ADHD. This early association appears to occur independently of ADHD subtype, suggesting that these conditions may follow distinct yet partially convergent developmental pathways, emphasizing the importance of identifying both shared and disorder-specific features [50].

In this context, a multidimensional assessment, including clinical interviews, questionnaires, behavioral observations, and objective tests, is essential [14]. Accurate differential diagnosis and recognition of comorbidities are crucial for informing treatment decisions.

A detailed anamnesis should be obtained, covering the individual’s developmental and psychiatric history and psychosocial factors [14,51]. Additionally, functional impairments across multiple life areas should be identified through an evaluation of mental state and the observation of behaviors and symptoms across various domains and settings. Relying solely on observer reports, such as the Child Behavior Checklist (CBCL) [52,53] and Conners’ rating scales [54,55,56,57], completed by parents and teachers, may not provide a complete picture of a child’s or adolescent’s behavioral difficulties. In contrast, structured and semi-structured diagnostic interviews, neuropsychological testing, and behavioral observations offer a broader understanding of the child’s clinical presentation. In particular, examining cognitive and executive functions can further clarify the individual’s strengths and weaknesses, with direct implications for treatment planning [14]. However, these methods are time- and cost-intensive and, therefore, not always feasible in clinical practice compared to observer reports and self-administered symptom questionnaires [58]. This practical limitation is further reflected in the literature, where considerable variability exists regarding the assessment of cognitive and executive functions. Despite its central role in ADHD and its comorbidities, the current literature lacks standardized guidelines indicating which tests should be preferred, and methodological differences limit comparability across studies [36].

In light of the above considerations, our primary aim is to address this gap by applying standardized and clinically reproducible tools to assess cognitive and executive functions—specifically attention, inhibition, and working memory—in children with ADHD, AD, and comorbid ADHD + AD.

Beyond this primary objective, this study also addresses the following secondary aims:

(a) To examine how clinical diagnoses of ADHD and AD are associated with distinct patterns of cognitive and executive functioning;
(b) To identify neuropsychological markers that distinguish ADHD from AD and ADHD + AD comorbidity;
(c) To investigate the relationship between ADHD and anxiety symptoms, specifically whether their co-occurrence results in overlapping or distinct cognitive, executive, and emotional profiles.

By addressing this methodological gap, we aim to promote more consistent assessment practices to enhance the diagnosis and clinical management of children with ADHD, AD, and comorbid ADHD + AD.

## 2. Materials and Methods

### 2.1. Participants

This study included 71 children and adolescents, aged 8 to 15 years, who were referred to the Neuropsychological Service of the Complex Operative Unit of Child Neuropsychiatry, Policlinico Umberto I, for learning difficulties, inattention, and distractibility in the two-year period of 2021–2022.

All children and parents underwent an initial interview with a child neuropsychiatrist who recorded the child’s history. Then, a complete neuropsychological battery and symptoms rating scales were administered to the patients by a team of neuropsychiatrists.

The inclusion criteria were an Intelligence Quotient (IQ) ≥ 85, as measured by the Italian version of Wechsler Intelligence Scale for Children Fourth Edition (WISC-IV) [59,60,61]; Italian as the native language; and parent-reported Child Behavior Checklist (CBCL/6–18 years, in its Italian form [52,62,63]) scores between 65 and 69 (“borderline”) or above 70 (“clinical”) on at least one DSM-oriented scale (anxiety problems; somatic problems; attention-deficit/hyperactivity problems; oppositional defiant problems; and conduct problems).

The exclusion criteria included the presence of depressive symptoms assessed by the Italian version of the Children Depression Inventory, Second-Edition (CDI-2) self-report [64,65,66] and other comorbidities (e.g., oppositional defiant disorder, conduct disorder). Additionally, those with borderline or pathological scores on the Affective Problems scale of the parent-reported CBCL were excluded.

The final sample included 48 children and adolescents (mean age 10.12 years; SD = 1.59 years) divided into three groups according to principal diagnosis based on the Diagnostic and Statistical Manual of Mental Disorder, Fifth Edition (DSM-5) criteria [7].

The demographic characteristics of the sample are shown in Table 1a. The distribution of the specific subtypes of ADHD and/or AD within each group is provided in Table 1b. In the ADHD + AD group, all participants were diagnosed with generalized anxiety disorder. Regarding school grades, 79% of the sample attended primary school, 19% attended junior high school, and the remaining 2% attended high school. None of the children and adolescents included in this study were taking medication for ADHD or for any anxiety-related disorders at the time of the assessment.

### 2.2. Ethical Considerations

The research was conducted in accordance with the ethical standards of the responsible ethics committee and the principles of the Declaration of Helsinki (1964) [67]. This work is part of a study approved with ethics approval reference 0543/2022 (6 July 2022) by CET (Territorial Ethics Committee Latium Area 1). A written and verbal informed consent form for use of the patients’ information and material for scientific purposes was signed by the patients and their parents/legal guardians in accordance with current practice in our institution. The informed consent was placed in the patients’ hospital chart. The data were collected retrospectively and stored in a password-protected database, ensuring strict anonymity.

### 2.3. Measures

The Wechsler Intelligence Scale for Children Fourth Edition (WISC-IV) [59,60,61] is a tool designed to assess the cognitive functioning of children and adolescents. It includes 10 core subtests and 4 supplementary subtests, which provide a measure of full-scale IQ (IQ) derived from four indices: Verbal Comprehension Index (VCI), Perceptual Reasoning Index (PRI), Working Memory Index (WMI), and Processing Speed Index (PSI) [61,68,69].

The VCI evaluates verbal abilities (e.g., vocabulary, comprehension, and general knowledge), based on the performance in Similarities, Vocabulary, and Comprehension subtests. The PRI measures non-verbal reasoning abilities (e.g., visuospatial abilities, perceptual organization, and problem-solving) and is derived from Block Design, Picture Concepts, and Matrix Reasoning subtests. The WMI measures the ability to temporarily store and manipulate information, using Digit Span and Letter–Number Sequencing subtests. PSI reflects the ability to efficiently and accurately perform visual–motor tasks, based on performance in the Coding and Symbol Search subtests [61,68,69].

Each index ranges from 40 to 160, with a mean of 100 and a standard deviation of 15. Higher scores indicate better performance. The WISC-IV and its four index scores demonstrate strong psychometric properties, with excellent validity and high reliability. Internal consistency is robust across all composite scores (≥0.88), reaching 0.97 for the full-scale IQ, while test–retest reliability ranges from 0.86 to 0.93 across the indices [68,70].

In our study, we considered only the IQ and the four composite index scores (VCI, PRI, WMI, and PSI) obtained from the administration of the 10 core subtests. Supplementary subtests were not administered, and individual subtest scores were not used for the analyses.

The NEPSY-II [71,72,73,74] is a comprehensive neuropsychological battery for children aged 3–16 years. The Italian version [73,75] consists of 33 subtests assessing six domains: attention and executive functioning, language, memory and learning, sensorimotor functioning, social perception, and visuospatial processing. The NEPSY-II demonstrates generally adequate to high test–retest reliability across many subtests and provides multiple sources of validity evidence (e.g., content, construct, concurrent, and clinical validity) [75].

For the purpose of this research, we focused on the attention and executive functioning domain, which encompasses subtests that assess inhibition of learned and automatic responses; monitoring and self-regulation; vigilance; selective and sustained attention; the capacity to establish, maintain, and change a response set; non-verbal problem-solving; and planning and organizing a complex response. Specifically, we selected three NEPSY-II subtests: Visual Attention, Auditory Attention, and Inhibition. Subtest selection was driven by the study’s focus on key cognitive functions: working memory, processing speed, attention, and inhibition. While the subtests were not identified a priori, they were carefully chosen during the analysis phase to align with the specific research goals.

Visual Attention measures the ability to quickly and accurately focus and sustain attention on target visual stimuli presented among distractors. Visual Accuracy is determined by the number of correctly identified target items minus the number of incorrectly reported distracting items. Low scores may indicate an attention deficit as well as a deficit in planning and attention shift. Auditory Attention assesses selective auditory attention and the ability to sustain it (vigilance). In this task, the child listens to a series of words and is instructed to tap the appropriate circle upon hearing a target word within 2 s. Correct responses within this time frame are awarded one point; responses after 2 s are considered omission errors and receive no points. Inhibition measures the ability to inhibit automatic responses and apply task-specific rules. The child is presented with a series of black and white shapes or arrows and must name either the shape, direction, or an alternate response, depending on the color of the shape or arrow, respectively, in three phases: condition A, also known as naming (IN-A); condition B of inhibition (IN-B); and condition C of switching (IN-C). This subtest is based on the Stroop (1935) paradigm, in which an automatic verbal response (e.g., reading words printed in colored ink) must be inhibited in order to produce a conflicting response (e.g., naming the ink color) [73]. The Inhibition subtest adapts this approach to a non-reading naming task. It is a timed measure designed to assess the ability to suppress automatic responses in favor of novel ones and to flexibly switch between different response types. Elevated scores are indicative of better performances [76].

The Multidimensional Anxiety Scale for Children-Second Edition (MASC 2) is a revised version of the original MASC, a self-report instrument for measuring anxiety symptoms in individuals aged 8 to 19 years [77,78,79,80]. The MASC 2 self-report form consists of 50 items evaluating emotional, physical, cognitive, and behavioral symptoms of anxiety. Each item of the scale is rated on a Likert scale ranging from 0 to 4. Scores are converted into standardized *T*-scores, which have a mean of 50 and a standard deviation of 10. The responses on the MASC 2 are combined to create 11 T-scores: MASC 2 Total Score, Separation, Anxiety/Phobias, Generalized Anxiety Disorder (GAD) Index, Social Anxiety (Total, Humiliation/Rejection, Performance Fears), Obsession and Compulsions, Physical Symptoms (Total, Tens/Restless, Panic), and Harm Avoidance. Higher T-scores indicate more severe and/or a greater number of symptoms. For scales/subscales, *T* scores below 60 are considered average, scores between 60 and 64 are considered slightly elevated, scores between 65 and 69 are considered elevated, and scores 70 and above are considered very elevated. The MASC 2 presents good internal consistency (Cronbach’s α = 0.89) and strong test–retest reliability (r = 0.89) for the total score [77,81]. It should be noted that, for the purpose of the present study, the total T-score of the MASC 2 was used as the primary indicator of overall anxiety symptom severity. Otherwise, subscale scores were specifically considered for the classification of AD subtypes in the participants within the AD and ADHD + AD groups.

The standardized Italian version of WISC-IV [59,61], NEPSY-II [76], and MASC 2 [79] were administered to all children and adolescents of the sample during the routine clinical activities of the Neuropsychological Center of Policlinico Umberto I Hospital of Rome.

### 2.4. Data Analysis

Data analysis was performed using the Jamovi software version 2.3.28 [82] and Excel. A descriptive analysis was first conducted to explore the distributions of the variables, followed by an inferential analysis, ensuring that the assumptions for each test were met. The assumption of homoscedasticity was assessed using Levene’s test, which indicated whether the distributions exhibited the same variance. If homoscedasticity was assumed, the Tukey test was applied to determine whether two or more distributions were statistically different. The assumptions for the Tukey test included independent observations within and between groups, a normal distribution of the groups associated with each test mean, and equal variance within and between groups (homogeneity of variance). In cases where homoscedasticity was not assumed, the non-parametric Nemenyi–Damico–Wolfe–Dunn test was used instead. To assess the strength and direction of the association between the variables of interest, Pearson’s correlation coefficient was calculated. The *t*-test for independent samples was applied to compare the means of two independent groups and to determine whether there was a statistically significant difference between them. To account for multiple comparisons in post hoc analyses, the Bonferroni correction was applied.

## 3. Results

The three groups were balanced for age and school grade. No significant differences in IQ were found across the diagnostic groups (see Supplementary File—Table S1). Likewise, there were no statistically significant differences in the WISC-IV indexes of VCI, PRI, WMI, and PSI.

**Figure 1:**
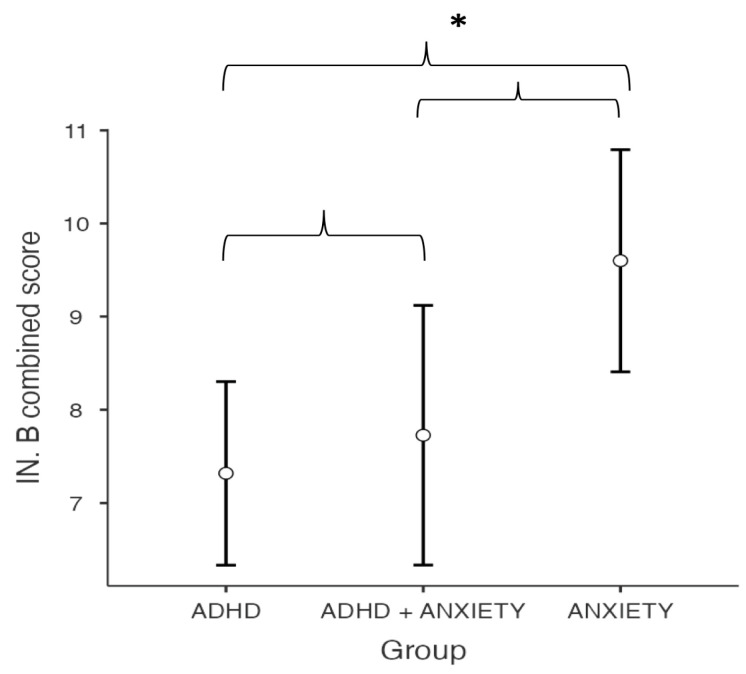
Between-group differences in IN-B combined score of the NEPSY-II Inhibition. Asterisks (*) denote statistically significant differences (*p* < 0.05).

The type of diagnosis has a significant effect on the combined score obtained in the IN-B inhibition task of the NEPSY-II (F(2,45) = 4.63, *p* = 0.015, η^2^p = 0.171). Post hoc comparisons between the means revealed a significantly higher score on the inhibition task for the anxiety group compared to the ADHD group (M_ADHD_ = 7.32, SE = 0.489 vs. M_AD_ = 7.73, SE = 0.691, p_tukey_ = 0.013, d = 0.995, 95% CI [0.28–1.70]). No statistically significant differences were found in the inhibition task between the ADHD group and the ADHD + AD group (p_tukey_ = 0.880, d = 0.178, 95% CI [0.56, 0.92]) or between the AD group and the ADHD + AD group (pt_ukey_ = 0.110, d = 0.817, 95% CI [0.00, 1.63]) (see Figure 1).

Similarly, the Tukey test indicated that the AD group completed the Inhibitory Control and Cognitive Flexibility subtest (IN-B) significantly faster than the ADHD group (*p* = 0.049). The IN-B subtest appeared to be most affected by the presence of ADHD, whether as a standalone condition or in comorbidity with AD. A statistically significant positive correlation (r(48) = 0.349) was observed between the IN-B combined score and the MASC 2 total score, with higher MASC 2 scores reported in both the AD group (mean: 66.5 ± 14.5) and the ADHD + AD group (mean: 60.2 ± 5.74).

No significant group differences were found in performance on the other NEPSY-II subtests, including conditions A and C of Inhibition (time, errors, and combined scores) as well as Visual Attention (targets and distractors) and Auditory Attention (correct target words and omission errors) (see Supplementary File—Tables S2–S5).

The Nemenyi–Damico–Wolfe–Dunn test applied to the MASC 2 self-report questionnaire total score showed that both the Anxiety group (*p* = 0.0015) and the ADHD + AD group (*p* = 0.00236) had significantly higher total scores compared to the ADHD group.

No significant correlations were found between sex and ADHD (*p* = 0.058) or AD (*p* = 0.151).

A significant positive correlation was found between IQ (measured by WISC-IV) and conditions A (r(48) = 0.394) and C (r(48) = 0.321) combined scores for Inhibition in the NEPSY-II. Specifically, the IN-A combined score was positively correlated with PRI (r(48) = 0.330) and WMI (r(48) = 0.412), while the IN-C combined score showed positive relationships with PRI (r(48) = 0.371), WMI (r(48) = 0.379), and PSI (r(48) = 0.296).

Significant correlations were found between errors on the IN-A and IN-C subtests of the NEPSY-II (expressed as scalar scores) and IQ, PRI, and WMI. Fewer errors in these subtests were associated with higher scalar scores, which, in turn, correlated with better performance on the WISC-IV, particularly on PRI, WMI, and overall IQ (see Table 2).

Omission errors on the Auditory Attention subtest of the NEPSY-II were negatively correlated with WMI on the WISC-IV (r(48) = −0.322), as shown in Table 2.

## 4. Discussion

The aim of the present study was to examine the cognitive and executive functions—specifically attention, inhibition, and working memory—in a clinical sample of school-age children and adolescents diagnosed with ADHD, AD, and ADHD + AD. These functions were evaluated using the standardized WISC-IV and NEPSY-II instruments, administered in a clinical setting.

Three key findings emerged from the present study. First, no significant differences were observed between groups in overall cognitive profiles, as assessed by the WISC-IV, or in executive functioning, as measured by the majority of NEPSY-II subtests. Second, significant group differences in inhibition performance (IN-B subtest) were found, with better performance in the AD group compared to the ADHD group and the ADHD + AD group. Third, correlation analyses revealed a positive association between anxiety symptoms and inhibition performance and a negative association between working memory and sustained attention errors.

These findings warrant further consideration in light of previous research. The absence of significant differences in overall cognitive profiles among the three groups is consistent with previous studies. Although both ADHD and AD may affect cognitive processes, these influences do not necessarily result in significant differences on standardized intelligence measures, such as the WISC-IV [83,84,85]. Individuals with ADHD often show slightly lower IQ scores and deficits in processing speed and working memory compared to typically developing peers; however, these differences are generally not clinically meaningful [86,87,88,89]. Similarly, both low and high IQ levels have been linked to anxiety disorders [83,90,91,92]. One study found that social and separation anxiety reported by parents and panic symptoms reported by youth were associated with lower IQ, whereas general anxiety symptoms were not related to IQ [92]. It should be noted that, despite consistency with prior research, our limited sample size may have reduced the power to detect subtle group differences. These findings, nonetheless, underscore that global cognitive ability alone is unlikely to distinguish between ADHD, AD, and comorbid presentations, highlighting the importance of assessing specific executive functions to better capture relevant differences.

In this regard, we observed a significant effect of ADHD diagnosis on performance, specifically in one NEPSY-II subtest (IN-B of inhibition). Inhibition is a critical executive function that supports various cognitive domains, including learning, motor control, and decision-making, particularly under time pressure [93,94,95,96].

Our findings showed that children with ADHD exhibited significantly poorer inhibition performance compared to those with AD, as reflected by longer response times and lower combined scores (time combined with errors) in the IN-B task. Inhibition deficits are a core feature of ADHD [24], closely linked to attentional and behavioral regulation difficulties [93,95,96,97]. Similarly, the AD group scored higher in the IN-B subtest for combined score. Since statistically significant differences were found only for time and combined scores but not for the number of errors, our findings suggest that time may be the key variable determining the differences between these groups. These results suggest that anxiety might influence behavioral regulation during tasks involving attentional and inhibitory control, whereas ADHD is associated with greater difficulties.

Conversely, the more efficient inhibition performance observed in the AD group may reflect compensatory strategies or increased task focus under time pressure. The observed association between inhibition performance and anxiety symptoms further highlights the complex interplay between AD and executive functioning. Some studies have shown that anxiety can hinder inhibition by affecting attentional control, making individuals more prone to distractions [4,98,99]. However, in our study, the children and adolescents in the AD group performed more efficiently on the IN-B task, particularly in terms of response speed. This finding suggests that anxiety may, in some contexts, promote greater task engagement and behavioral control during time-constrained tasks. Nevertheless, this interpretation remains speculative, and the interaction between ADHD and anxiety on inhibition performance warrants further investigation.

Regarding the ADHD + AD comorbidity group, no statistically significant differences were found between this group and the others for any NEPSY-II subtests, making it difficult to draw firm conclusions regarding whether these individuals follow the ADHD or AD executive performance pattern. Given the limited sample size, these findings should be interpreted with caution, as the absence of significant differences may reflect insufficient statistical power rather than true similarity across groups, and the question remains somewhat controversial. A recent meta-analysis [100] found that children with ADHD + AD performed better on inhibition tasks than those with ADHD alone, though this was limited to medication-naïve participants. Similarly, our study did not find greater attention or working memory deficits in children with ADHD + AD compared to those with ADHD alone. However, this result does not provide evidence to support or refute the hypothesis that AD might serve as a protective factor for inhibition deficits in ADHD [100,101].

The existing literature on the influence of AD on executive functioning in ADHD remains conflicting. Three independent reviews on this topic have concluded that AD in individuals with ADHD could be both a risk and a protective factor for executive dysfunctions [102,103,104]. While anxiety may negatively affect attention and working memory, it may also reduce impulsivity and improve response inhibition. Although our study did not explicitly test this hypothesis and did not provide conclusive evidence to support or refute the protective role of anxiety, the present findings are consistent with previous research suggesting that anxiety could modulate certain aspects of executive functioning, such as rule maintenance, stimulus detection, and action selection [105,106]. However, the interaction between ADHD and AD remains complex and warrants further investigation.

The observed positive association between inhibition performance and anxiety symptoms further highlights the complex interplay between AD and executive functioning. A Pearson correlation analysis revealed a significant correlation between the IN-B combined score and the MASC 2 total score, suggesting that greater anxiety symptoms are linked to better performance on inhibition tasks. This finding is consistent with the research conducted by Murphy [107], who found that youths with significant anxiety symptoms exhibited poorer working memory and longer response times but showed better inhibition compared to those with minimal or no anxiety symptoms. These results support the idea that anxiety might counterbalance some of the cognitive difficulties seen in ADHD, particularly in terms of impulsivity and behavioral regulation [105,106,108]. Several studies have also suggested a specific relationship between anxiety and ADHD-inattentive type [109], but these results remain controversial [110]. However, this relationship was not analyzed in our study, since we did not consider the type of AD. The interaction between ADHD and AD is widely debated in the literature, since they have independent transmission patterns, represent different expressions of the same genetic risk factor [48,111], and share some neurodevelopmental brain abnormalities [106,112,113,114].

In addition to inhibition, our results showed a negative correlation between working memory and errors on the Auditory Attention subtest, which assesses sustained auditory attention. Working memory is typically described as a cognitive framework for the temporary storage and manipulation of information [115]. Poor working memory is a common neuropsychological feature in children and adolescents with ADHD or anxiety. In children with ADHD, impaired working memory contributes to persistent difficulties in anticipating, planning, executing, and maintaining goal-directed actions [26,116]. Children with ADHD often show deficits in vigilance, selective and divided attention, cognitive flexibility, and both phonological and visuospatial working memory [117,118]. Perseverative errors in children with ADHD may stem from an interaction between behavioral inhibition and working memory. These children often fail to retain information about the success or failure of their previous responses, which can influence or disrupt subsequent decisions [24,119]. Children with more severe AD, and a higher number of anxiety diagnoses, tend to show poorer performance in memory (particularly visuospatial and semantic memory) and language tasks compared to children with less severe anxiety [120,121,122,123]. The co-occurrence of anxiety in children with ADHD does not appear to alter working memory performance, as demonstrated by several studies [124,125,126,127,128,129,130]. However, anxiety seems to modulate the relationship between ADHD severity and neural activity in regions such as the cerebellum (for working memory contrasts) and in the striatum and thalamus (for memory load contrasts). The involvement of these brain regions suggests that the combined presence of ADHD and anxiety may affect the filtering of information, while at lower levels of anxiety, other mechanisms might contribute to any negative relationship between ADHD severity and working memory performance. These interaction effects could help explain the heterogeneity observed in studies of ADHD and anxiety, which often produce inconsistent findings [131]. Our results suggest that anxiety in children with ADHD does not necessarily exacerbate working memory impairments, as has been reported in some previous studies.

Taken together, these findings contribute to the growing understanding of the complex interplay between ADHD, anxiety, and cognitive and executive functioning. However, the present study has several limitations. Firstly, the small sample size limited the ability to conduct in-depth analyses and reduced the generalizability of our findings. This limitation also affects the statistical power to detect significant differences between groups for the WISC-IV indexes and the other NEPSY-II subtests. Additionally, executive functions were assessed only using the NEPSY-II, which precludes further correlations with other testing methods and limits the ability to explore other cognitive functions, such as planning. Given the relatively small sample size for each diagnostic group, more detailed analyses regarding specific ADHD subtypes and the different subtypes of AD were not feasible. Consequently, while these subtypes were considered in group classification, more granular analyses would require a larger sample size to ensure adequate statistical power and reliability. Future research should include larger samples, consider ADHD and AD subtypes and gender effects, and further explore the clinical implications of these cognitive and executive function profiles to guide personalized interventions.

## 5. Conclusions

The present study demonstrates that children with ADHD exhibit poorer inhibition compared to those with AD. This is reflected by longer response times, likely resulting from attention deficits and difficulties in behavioral control. This finding may suggest that the effects of ADHD and AD on inhibitory control are distinct when the two conditions are not comorbid. Furthermore, the observed correlation between inhibition and anxiety symptoms underscores the importance of a comprehensive assessment approach, particularly when ADHD symptomatology overlaps with that of AD. Integrating cognitive, executive, and emotional evaluation tools may help clinicians improve diagnostic accuracy. In this context, this study supports the routine use of the inhibition subtest from the NEPSY-II, in combination with the MASC 2 questionnaire, to facilitate differential diagnosis in clinical practice.

## Figures and Tables

**Table 1:** (**a**) Demographic characteristics of the sample. M: male, F: female. (**b**) Distribution of the specific subtypes of ADHD and/or AD within each group. M: male, F: female, GAD: generalized anxiety disorder.

(a)			
Group	Participants	Mean Age (years)	Gender
ADHD	22	9.9 (±1.17)	M = 12, F = 10
AD	15	10.52 (±1.91)	M = 10, F = 5
ADHD + AD	11	9.84 (±1.61)	M = 8, F = 3
Total	48	10.12 (±1.59)	M = 30, F = 18
**(b)**			
**Group**		**Subtypes**	**Participants**
ADHD		Inattentive	8 (M = 4, F = 4)
		Hyperactive/Impulsive	5 (M = 5)
		Combined	9 (M = 3, F = 6)
AD		GAD	10 (M = 7, F = 3)
		Separation	4 (M = 3, F = 1)
		Social	1 (F = 1)
ADHD + AD		Inattentive + GAD	5 (M = 3, F = 2)
		Hyperactive/Impulsive + GAD	2 (M = 2)
		Combined + GAD	4 (M = 3, F = 1)

**Table 2:** Correlation coefficients between WISC-IV indexes and NEPSY-II subtests.

NEPSY-II Subtest and Score	IQ	PRI	WMI	PSI
Auditory Attention: Omission Error	−0.274	−0.128	−0.322 *	0.033
Naming (IN-A): errors	0.282	0.331 *	0.329 *	0.038
Naming (IN-A): combined scalar score	0.394 **	0.330 *	0.412 **	0.106
Switching (IN-C): errors	0.317 *	0.471 **	0.350 *	0.239
Switching (IN-C): combined scalar score	0.321 *	0.371 *	0.379 **	0.296 *

** Correlation is significant at the 0.01 level (2-tailed). * Correlation is significant at the 0.05 level (2-tailed).

## Data Availability

The data presented in this study are available on request from the corresponding author due to privacy restrictions.

## Supplementary Materials

The following supporting information can be downloaded at: https://www.mdpi.com/article/10.3390/children12060798/s1, Table S1: Between-group differences in cognitive function measured through WISC-IV, Table S2: Between-group differences in Naming (NEPSY-II Inhibition subtest, condition IN-A), Table S3: Between-group differences in Switching (NEPSY-II Inhibition subtest, condition IN-C), Table S4: Between-group differences in Visual Attention (NEPSY-II), Table S5: Between-group differences in Auditory Attention (NEPSY-II).

## Abbreviations

The following abbreviations are used in this manuscript:

ADHD	Attention Deficit Hyperactivity Disorder
AD	Anxiety Disorders
SLDs	Specific Learning Disorders
CBCL	The Child Behavior Checklist
IQ	Intelligence Quotient
WISC-IV	The Wechsler Intelligence Scale for Children Fourth Edition
DSM-5	Diagnostic and Statistical Manual of Mental Disorders Fifth Edition
CDI-2	The Children Depression Inventory Second Edition
VCI	Verbal Comprehension Index
PRI	Perceptual Reasoning Index
WMI	Working Memory Index
PSI	Processing Speed Index
IN	Inhibition
IN-A	Naming, Condition A of Inhibition
IN-B	Inhibition, Condition B of Inhibition
IN-C	Switching, Condition C of Inhibition
MASC 2	The Multidimensional Anxiety Scale for Children-Second Edition

## References

[1] Berggren N., Derakshan N. (2013). Attentional Control Deficits in Trait Anxiety: Why You See Them and Why You Don’t. Biol. Psychol..

[2] Crosbie J., Arnold P., Paterson A., Swanson J., Dupuis A., Li X., Shan J., Goodale T., Tam C., Strug L.J. (2013). Response Inhibition and ADHD Traits: Correlates and Heritability in a Community Sample. J. Abnorm. Child Psychol..

[3] Epstein J.N., Erkanli A., Conners C.K., Klaric J., Costello J.E., Angold A. (2003). Relations Between Continuous Performance Test Performance Measures and ADHD Behaviors. J. Abnorm. Child Psychol..

[4] Eysenck M.W., Derakshan N., Santos R., Calvo M.G. (2007). Anxiety and Cognitive Performance: Attentional Control Theory. Emotion.

[5] Xia L., Mo L., Wang J., Zhang W., Zhang D. (2020). Trait Anxiety Attenuates Response Inhibition: Evidence From an ERP Study Using the Go/NoGo Task. Front. Behav. Neurosci..

[6] Sayal K., Prasad V., Daley D., Ford T., Coghill D. (2018). ADHD in Children and Young People: Prevalence, Care Pathways, and Service Provision. Lancet Psychiatry.

[7] American Psychiatric Association (2013). Diagnostic and Statistical Manual of Mental Disorders (DSM-5).

[8] Hart E.L., Lahey B.B., Loeber R., Applegate B., Frick P.J. (1995). Developmental Change in Attention-Deficit Hyperactivity Disorder in Boys: A Four-Year Longitudinal Study. J. Abnorm. Child Psychol..

[9] Lara C., Fayyad J., De Graaf R., Kessler R.C., Aguilar-Gaxiola S., Angermeyer M., Demytteneare K., De Girolamo G., Haro J.M., Jin R. (2009). Childhood Predictors of Adult Attention-Deficit/Hyperactivity Disorder: Results from the World Health Organization World Mental Health Survey Initiative. Biol. Psychiatry.

[10] Biederman J., Petty C.R., Clarke A., Lomedico A., Faraone S.V. (2011). Predictors of Persistent ADHD: An 11-Year Follow-up Study. J. Psychiatr. Res..

[11] Kessler R.C., Green J.G., Adler L.A., Barkley R.A., Chatterji S., Faraone S.V., Finkelman M., Greenhill L.L., Gruber M.J., Jewell M. (2010). Structure and Diagnosis of Adult Attention-Deficit/Hyperactivity Disorder: Analysis of Expanded Symptom Criteria From the Adult ADHD Clinical Diagnostic Scale. Arch. Gen. Psychiatry.

[12] Faraone S.V., Biederman J., Mick E. (2006). The Age-Dependent Decline of Attention Deficit Hyperactivity Disorder: A Meta-Analysis of Follow-up Studies. Psychol. Med..

[13] Larsson H., Lichtenstein P., Larsson J.-O. (2006). Genetic Contributions to the Development of ADHD Subtypes From Childhood to Adolescence. J. Am. Acad. Child Adolesc. Psychiatry.

[14] Sadr-Salek S., Costa A.P., Steffgen G. (2023). Psychological Treatments for Hyperactivity and Impulsivity in Children with ADHD: A Narrative Review. Children.

[15] Racz S.J., Putnick D.L., Suwalsky J.T.D., Hendricks C., Bornstein M.H. (2017). Cognitive Abilities, Social Adaptation, and Externalizing Behavior Problems in Childhood and Adolescence: Specific Cascade Effects Across Development. J. Youth Adolesc..

[16] Kawabata Y., Tseng W.-L., Gau S.S.-F. (2012). Symptoms of Attention-Deficit/Hyperactivity Disorder and Social and School Adjustment: The Moderating Roles of Age and Parenting. J. Abnorm. Child Psychol..

[17] Franke B., Michelini G., Asherson P., Banaschewski T., Bilbow A., Buitelaar J.K., Cormand B., Faraone S.V., Ginsberg Y., Haavik J. (2018). Live Fast, Die Young? A Review on the Developmental Trajectories of ADHD across the Lifespan. Eur. Neuropsychopharmacol..

[18] Nigg J.T., Goldsmith H.H., Sachek J. (2004). Temperament and Attention Deficit Hyperactivity Disorder: The Development of a Multiple Pathway Model. J. Clin. Child Adolesc. Psychol..

[19] Cheung C.H.M., Rijdijk F., McLoughlin G., Faraone S.V., Asherson P., Kuntsi J. (2015). Childhood Predictors of Adolescent and Young Adult Outcome in ADHD. J. Psychiatr. Res..

[20] Poznyak E., Debbané M. (2025). Emotion Regulation beyond Executive and Attention Difficulties: Impact on Daily Life Impairments in Community Adolescents. Child Adolesc. Psychiatry Ment. Health.

[21] Duff C.T., Sulla E.M. (2015). Measuring Executive Function in the Differential Diagnosis of Attention-Deficit/Hyperactivity Disorder: Does It Really Tell Us Anything?. Appl. Neuropsychol. Child.

[22] Jensen P.S., Hinshaw S.P., Kraemer H.C., Lenora N., Newcorn J.H., Abikoff H.B., March J.S., Arnold L.E., Cantwell D.P., Conners C.K. (2001). ADHD Comorbidity Findings from the MTA Study: Comparing Comorbid Subgroups. J. Am. Acad. Child Adolesc. Psychiatry.

[23] Carroll J.M., Maughan B., Goodman R., Meltzer H. (2005). Literacy Difficulties and Psychiatric Disorders: Evidence for Comorbidity. J. Child Psychol. Psychiatry.

[24] Barkley R.A. (1997). Behavioral Inhibition, Sustained Attention, and Executive Functions: Constructing a Unifying Theory of ADHD. Psychol. Bull..

[25] Rapport M.D., Chung K.-M., Shore G., Isaacs P. (2001). A Conceptual Model of Child Psychopathology: Implications for Understanding Attention Deficit Hyperactivity Disorder and Treatment Efficacy. J. Clin. Child Adolesc. Psychol..

[26] Kofler M.J., Sarver D.E., Harmon S.L., Moltisanti A., Aduen P.A., Soto E.F., Ferretti N. (2018). Working Memory and Organizational Skills Problems in ADHD. J. Child Psychol. Psychiatry.

[27] Halperin J.M., Schulz K.P. (2006). Revisiting the Role of the Prefrontal Cortex in the Pathophysiology of Attention-Deficit/Hyperactivity Disorder. Psychol. Bull..

[28] Van Lieshout M., Luman M., Buitelaar J., Rommelse N.N.J., Oosterlaan J. (2013). Does Neurocognitive Functioning Predict Future or Persistence of ADHD? A Systematic Review. Clin. Psychol. Rev..

[29] Ferreri F., Lapp L.K., Peretti C.-S. (2011). Current Research on Cognitive Aspects of Anxiety Disorders. Curr. Opin. Psychiatry.

[30] Hirsch C.R., Mathews A. (2012). A Cognitive Model of Pathological Worry. Behav. Res. Ther..

[31] Alfonso S.V., Lonigan C.J. (2021). Trait Anxiety and Adolescent’s Academic Achievement: The Role of Executive Function. Learn. Individ. Differ..

[32] Moran T.P. (2016). Anxiety and Working Memory Capacity: A Meta-Analysis and Narrative Review. Psychol. Bull..

[33] Ursache A., Raver C.C. (2014). Trait and State Anxiety: Relations to Executive Functioning in an at-Risk Sample. Cogn. Emot..

[34] Wright L., Lipszyc J., Dupuis A., Thayapararajah S.W., Schachar R. (2014). Response Inhibition and Psychopathology: A Meta-Analysis of Go/No-Go Task Performance. J. Abnorm. Psychol..

[35] Comer J.S., Blanco C., Hasin D.S., Liu S.-M., Grant B.F., Turner J.B., Olfson M. (2011). Health-Related Quality of Life Across the Anxiety Disorders: Results From the National Epidemiologic Survey on Alcohol and Related Conditions (NESARC). J. Clin. Psychiatry.

[36] Marsh C.L., Harmon S.L., Cho S., Chan E.S.M., Gaye F., DeGeorge L., Black K.E., Irwin Harper L.N., Kofler M.J. (2024). Does Anxiety Systematically Bias Estimates of Executive Functioning Deficits in Pediatric Attention-Deficit/Hyperactivity Disorder?. Res. Child Adolesc. Psychopathol..

[37] Brown T.E., Buitelaar J.K., Kan C.C., Asherson P. (2011). Adult ADHD and Mood Disorders. ADHD in Adults.

[38] D’Agati E., Curatolo P., Mazzone L. (2019). Comorbidity between ADHD and Anxiety Disorders across the Lifespan. Int. J. Psychiatry Clin. Pract..

[39] Sadek J. (2019). Clinician’s Guide to ADHD Comorbidities in Children and Adolescents: Case Studies.

[40] Koyuncu A., Ince E., Ertekin E., Çelebi F., Tükel R. (2019). Is There a Prodrom Period in Patients with Social Anxiety Disorder? A Discussion on the Hypothesis of Social Anxiety Disorder Development Secondary to Attention-Deficit/Hyperactivity Disorder. ADHD Atten. Deficit Hyperact. Disord..

[41] Tai Y.-M., Gau C.-S., Gau S.S.-F., Chiu H.-W. (2013). Prediction of ADHD to Anxiety Disorders: An 11-Year National Insurance Data Analysis in Taiwan. J. Atten. Disord..

[42] Souza I., Pinheiro M., Denardin D., Mattos P., Rohde L. (2004). Attention-Deficit/Hyperactivity Disorder and Comorbidity in Brazil: Comparisons between Two Referred Samples. Eur. Child Adolesc. Psychiatry.

[43] You Y., Oginni O.A., Rijsdijk F.V., Lim K.X., Zavos H.M.S., McAdams T.A. (2024). Exploring Associations between ADHD Symptoms and Emotional Problems from Childhood to Adulthood: Shared Aetiology or Possible Causal Relationship?. Psychol. Med..

[44] Roth R.M., Wishart H.A., Flashman L.A., Riordan H.J., Huey L., Saykin A.J. (2004). Contribution of Organizational Strategy to Verbal Learning and Memory in Adults With Attention-Deficit/Hyperactivity Disorder. Neuropsychology.

[45] Pliszka S.R. (2000). Patterns of Psychiatric Comorbidity with Attention-Deficit/Hyperactivity Disorder. Child Adolesc. Psychiatr. Clin. N. Am..

[46] Manassis K., Tannock R., Young A., Francis-John S. (2007). Cognition in anxious children with attention deficit hyperactivity disorder: A comparison with clinical and normal children. Behav. Brain Funct..

[47] Gnanavel S., Sharma P., Kaushal P., Hussain S. (2019). Attention Deficit Hyperactivity Disorder and Comorbidity: A Review of Literature. World J. Clin. Cases.

[48] Ghirardi L., Pettersson E., Taylor M.J., Freitag C.M., Franke B., Asherson P., Larsson H., Kuja-Halkola R. (2019). Genetic and Environmental Contribution to the Overlap between ADHD and ASD Trait Dimensions in Young Adults: A Twin Study. Psychol. Med..

[49] Plourde V., Boivin M., Brendgen M., Vitaro F., Dionne G. (2017). Phenotypic and Genetic Associations between Reading and Attention-Deficit/Hyperactivity Disorder Dimensions in Adolescence. Dev. Psychopathol..

[50] Overgaard K.R., Aase H., Torgersen S., Zeiner P. (2016). Co-Occurrence of ADHD and Anxiety in Preschool Children. J. Atten. Disord..

[51] Eom T.H., Kim Y.-H. (2024). Clinical Practice Guidelines for Attention-Deficit/Hyperactivity Disorder: Recent Updates. Clin. Exp. Pediatr..

[52] Achenbach T.M. (1999). The Child Behavior Checklist and Related Instruments. The Use of Psychological Testing for Treatment Planning and Outcomes Assessment.

[53] Achenbach T.M., Rescorla L.A. (2001). Manual for the ASEBA School-Age Forms & Profiles.

[54] Conners C.K. (1999). Conners Rating Scales-Revised. The Use of Psychological Testing for Treatment Planning and Outcomes Assessment.

[55] Conners C.K. (1997). Conners’ Rating Scales—Revised Technical Manual.

[56] Conners C.K., Erhardt D., Sparrow M.A. (1999). Conners Adult ADHD Rating Scales (CAARS).

[57] Conners C.K. (2008). Conners 3rd Edition: Manual.

[58] Pelham W.E., Fabiano G.A., Massetti G.M. (2005). Evidence-Based Assessment of Attention Deficit Hyperactivity Disorder in Children and Adolescents. J. Clin. Child Adolesc. Psychol..

[59] Orsini A., Pezzuti L., Picone L. (2012). WISC-IV: Contributo Alla Taratura Italiana.

[60] Cornoldi C., Orsini A., Cianci L., Giofrè D., Pezzuti L. (2013). Intelligence and Working Memory Control: Evidence from the WISC-IV Administration to Italian Children. Learn. Individ. Differ..

[61] Wechsler D., Rust J., Golombok S. (2004). WISC-IV Wechsler Intelligence Scale for Children.

[62] Frigerio A., Cattaneo C., Cataldo M., Schiatti A., Molteni M., Battaglia M. (2004). Behavioral and Emotional Problems Among Italian Children and Adolescents Aged 4 to 18 Years as Reported by Parents and Teachers. Eur. J. Psychol. Assess..

[63] d’Orlando F., Grassi M., Di Blas L. (2010). Uno studio di validazione del CBCL/6-18 e del TRF/6-18 nella tarda infanzia. G. Ital. Psicol..

[64] Kovacs M., Cautin R.L., Lilienfeld S.O. (2015). Children’s Depression Inventory (CDI and CDI 2). The Encyclopedia of Clinical Psychology.

[65] Kovacs M. (2018). Children’s Depression Inventory.

[66] Bae Y. (2012). Test Review: Children’s Depression Inventory 2 (CDI 2). J. Psychoeduc. Assess..

[67] World Medical Association (2013). World Medical Association Declaration of Helsinki: Ethical Principles for Medical Research Involving Human Subjects. JAMA.

[68] Canivez G.L. (2014). Construct Validity of the WISC-IV with a Referred Sample: Direct versus Indirect Hierarchical Structures. Sch. Psychol. Q..

[69] Watkins M.W. (2010). Structure of the Wechsler Intelligence Scale for Children—Fourth Edition among a National Sample of Referred Students. Psychol. Assess..

[70] Kaufman A.S., Flanagan D.P., Alfonso V.C., Mascolo J.T. (2006). Test Review: Wechsler Intelligence Scale for Children, Fourth Edition (WISC-IV). J. Psychoeduc. Assess..

[71] Korkman M., Kirk U., Kemp S. (2012). NEPSY.

[72] Korkman M. (1998). NEPSY: A Developmental Neuropsychological Assessment Manual.

[73] Korkman M., Kirk U., Kemp S., Urgesi C., Campanella F., Fabbro F. (2015). NEPSY-II.

[74] Korkman M., Pesonen A.-E. (1994). A Comparison of Neuropsychological Test Profiles of Children with Attention Deficit—Hyperactivity Disorder and/or Learning Disorder. J. Learn. Disabil..

[75] Brooks B.L., Sherman E.M.S., Strauss E. (2009). NEPSY-II: A Developmental Neuropsychological Assessment, Second Edition. Child Neuropsychol..

[76] Urgesi C., Campanella F., Fabbro F. (2011). NEPSY-II. Contributo Alla Taratura Italiana.

[77] March J.S., Paloscia C., Giangregorio A., Guerini R., Melchiori F.M. (2017). Masc 2 TM. Multidimensional Anxiety Scale for Children.

[78] March J.S., Parker J.D.A., Maruish M.E. (2004). The Multidimensional Anxiety Scale for Children (MASC). The Use of Psychological Testing for Treatment Planning and Outcomes Assessment: Instruments for Children and Adolescents.

[79] Fraccaro R.L., Stelnicki A.M., Nordstokke D.W. (2015). Test Review: Multidimensional Anxiety Scale for Children by J. S. March. Can. J. Sch. Psychol..

[80] Melchiori F., Paloscia C., Giangregorio A., Guerini R. (2017). Manual of Multidimensional Anxiety Scale Fo Children Second Edition 2—Italian Version.

[81] Baldwin J.S., Dadds M.R. (2007). Reliability and Validity of Parent and Child Versions of the Multidimensional Anxiety Scale for Children in Community Samples. J. Am. Acad. Child Adolesc. Psychiatry.

[82] The Jamovi Project (2022). Jamovi.

[83] Ravagnani Salto A.B., Salum G.A., Hoffmann M.S., Santoro M.L., Zugman A., Pan P.M., Belangero S.I., Ito L.T., Doretto V.F., Croci M.S. (2025). The Trajectory of Anxiety Symptoms during the Transition from Childhood to Young Adulthood Is Predicted by IQ and Sex, but Not Polygenic Risk Scores. JCPP Adv..

[84] Agnew-Blais J.C., Polanczyk G.V., Danese A., Wertz J., Moffitt T.E., Arseneault L. (2020). Are Changes in ADHD Course Reflected in Differences in IQ and Executive Functioning from Childhood to Young Adulthood?. Psychol. Med..

[85] Hare C., Leslie A.C., Bodell L.P., Kaufman E.A., Morton J.B., Nicolson R., Kelley E., Jones J., Ayub M., Crosbie J. (2024). Sex and Intelligence Quotient Differences in Age of Diagnosis among Youth with Attention-deficit Hyperactivity Disorder. Br. J. Clin. Psychol..

[86] Bridgett D.J., Walker M.E. (2006). Intellectual Functioning in Adults with ADHD: A Meta-Analytic Examination of Full Scale IQ Differences between Adults with and without ADHD. Psychol. Assess..

[87] Frazier T.W., Demaree H.A., Youngstrom E.A. (2004). Meta-Analysis of Intellectual and Neuropsychological Test Performance in Attention-Deficit/Hyperactivity Disorder. Neuropsychology.

[88] Milioni A.L.V., Chaim T.M., Cavallet M., De Oliveira N.M., Annes M., Dos Santos B., Louzã M., Da Silva M.A., Miguel C.S., Serpa M.H. (2017). High IQ May “Mask” the Diagnosis of ADHD by Compensating for Deficits in Executive Functions in Treatment-Naïve Adults With ADHD. J. Atten. Disord..

[89] Antshel K.M., Faraone S.V., Stallone K., Nave A., Kaufmann F.A., Doyle A., Fried R., Seidman L., Biederman J. (2007). Is Attention Deficit Hyperactivity Disorder a Valid Diagnosis in the Presence of High IQ? Results from the MGH Longitudinal Family Studies of ADHD. J. Child Psychol. Psychiatry.

[90] Kermarrec S., Attinger L., Guignard J.-H., Tordjman S. (2020). Anxiety Disorders in Children with High Intellectual Potential. BJPsych Open.

[91] Melby L., Indredavik M.S., Løhaugen G., Brubakk A.M., Skranes J., Vik T. (2020). Is There an Association between Full IQ Score and Mental Health Problems in Young Adults? A Study with a Convenience Sample. BMC Psychol..

[92] Mahony B.W., Tu D., Rau S., Liu S., Lalonde F.M., Alexander-Bloch A.F., Satterthwaite T.D., Shinohara R.T., Bassett D.S., Milham M.P. (2023). IQ Modulates Coupling Between Diverse Dimensions of Psychopathology in Children and Adolescents. J. Am. Acad. Child Adolesc. Psychiatry.

[93] Engle R.W., Kane M.J., Ross B.H. (2004). Executive Attention, Working Memory Capacity, and a Two-Factor Theory of Cognitive Control. The Psychology of Learning and Motivation: Advances in Research and Theory.

[94] Howard S.J., Johnson J., Pascual-Leone J. (2014). Clarifying Inhibitory Control: Diversity and Development of Attentional Inhibition. Cogn. Dev..

[95] Engle R.W. (2018). Working Memory and Executive Attention: A Revisit. Perspect. Psychol. Sci..

[96] Diamond A. (2013). Executive Functions. Annu. Rev. Psychol..

[97] Miyake A., Friedman N.P., Emerson M.J., Witzki A.H., Howerter A., Wager T.D. (2000). The Unity and Diversity of Executive Functions and Their Contributions to Complex “Frontal Lobe” Tasks: A Latent Variable Analysis. Cognit. Psychol..

[98] Eysenck M.W., Derakshan N. (2011). New Perspectives in Attentional Control Theory. Personal. Individ. Differ..

[99] Derakshan N., Eysenck M.W. (2009). Anxiety, Processing Efficiency, and Cognitive Performance: New Developments from Attentional Control Theory. Eur. Psychol..

[100] Maric M., Bexkens A., Bögels S.M. (2018). Is Clinical Anxiety a Risk or a Protective Factor for Executive Functioning in Youth with ADHD? A Meta-Regression Analysis. Clin. Child Fam. Psychol. Rev..

[101] Klymkiw D.F., Milligan K., Lackner C., Phillips M., Schmidt L.A., Segalowitz S.J. (2020). Does Anxiety Enhance or Hinder Attentional and Impulse Control in Youth With ADHD? An ERP Analysis. J. Atten. Disord..

[102] Pliszka S.R. (1989). Effect of Anxiety on Cognition, Behavior, and Stimulant Response in ADHD. J. Am. Acad. Child Adolesc. Psychiatry.

[103] Schatz D.B., Rostain A.L. (2006). ADHD With Comorbid Anxiety: A Review of the Current Literature. J. Atten. Disord..

[104] Toplak M.E., Bucciarelli S.M., Jain U., Tannock R. (2008). Executive Functions: Performance-Based Measures and the Behavior Rating Inventory of Executive Function (BRIEF) in Adolescents with Attention Deficit/Hyperactivity Disorder (ADHD). Child Neuropsychol..

[105] Carlson C.L., Mann M. (2002). Sluggish Cognitive Tempo Predicts a Different Pattern of Impairment in the Attention Deficit Hyperactivity Disorder, Predominantly Inattentive Type. J. Clin. Child Adolesc. Psychol..

[106] Jarrett M.A., Ollendick T.H. (2008). A Conceptual Review of the Comorbidity of Attention-Deficit/Hyperactivity Disorder and Anxiety: Implications for Future Research and Practice. Clin. Psychol. Rev..

[107] Murphy Y.E., Luke A., Brennan E., Francazio S., Christopher I., Flessner C.A. (2018). An Investigation of Executive Functioning in Pediatric Anxiety. Behav. Modif..

[108] Menghini D., Armando M., Calcagni M., Napolitano C., Pasqualetti P., Sergeant J.A., Pani P., Vicari S. (2018). The Influence of Generalized Anxiety Disorder on Executive Functions in Children with ADHD. Eur. Arch. Psychiatry Clin. Neurosci..

[109] Biederman J., Monuteaux M.C., Mick E., Spencer T., Wilens T.E., Silva J.M., Snyder L.E., Faraone S.V. (2006). Young Adult Outcome of Attention Deficit Hyperactivity Disorder: A Controlled 10-Year Follow-up Study. Psychol. Med..

[110] Ghanizadeh A. (2008). Comorbidity of Psychiatric Disorders in Children and Adolescents with Alopecia Areata in a Child and Adolescent Psychiatry Clinical Sample. Int. J. Dermatol..

[111] Braaten E.B., Beiderman J., Monuteaux M.C., Mick E., Calhoun E., Cattan G., Faraone S.V. (2003). Revisiting the Association between Attention-Deficit/Hyperactivity Disorder and Anxiety Disorders: A Familial Risk Analysis. Biol. Psychiatry.

[112] Hilbert K., Evens R., Isabel Maslowski N., Wittchen H.-U., Lueken U. (2015). Neurostructural Correlates of Two Subtypes of Specific Phobia: A Voxel-Based Morphometry Study. Psychiatry Res. Neuroimaging.

[113] Semrud-Clikeman M., Pliszka S.R., Bledsoe J., Lancaster J. (2014). Volumetric MRI Differences in Treatment Naïve and Chronically Treated Adolescents With ADHD-Combined Type. J. Atten. Disord..

[114] Strawn J.R., Lu L., Peris T.S., Levine A., Walkup J.T. (2021). Research Review: Pediatric Anxiety Disorders—What Have We Learnt in the Last 10 Years?. J. Child Psychol. Psychiatry.

[115] Baddeley A. (2012). Working Memory: Theories, Models, and Controversies. Annu. Rev. Psychol..

[116] Kofler M.J., Sarver D.E., Wells E.L. (2020). Working Memory and Increased Activity Level (Hyperactivity) in ADHD: Experimental Evidence for a Functional Relation. J. Atten. Disord..

[117] Pasini A., Paloscia C., Alessandrelli R., Porfirio M.C., Curatolo P. (2007). Attention and Executive Functions Profile in Drug Naive ADHD Subtypes. Brain Dev..

[118] Tucha O., Walitza S., Mecklinger L., Sontag T.-A., Kübber S., Linder M., Lange K.W. (2006). Attentional Functioning in Children with ADHD—Predominantly Hyperactive-Impulsive Type and Children with ADHD—Combined Type. J. Neural Transm..

[119] Fuster J.M. (1991). Chapter 10 The Prefrontal Cortex and Its Relation to Behavior. Progress in Brain Research.

[120] Aronen E.T., Vuontela V., Steenari M.-R., Salmi J., Carlson S. (2005). Working Memory, Psychiatric Symptoms, and Academic Performance at School. Neurobiol. Learn. Mem..

[121] Sbicigo J.B., Toazza R., Becker N., Ecker K., Manfro G.G., Salles J.F.D. (2020). Memory and Language Impairments Are Associated with Anxiety Disorder Severity in Childhood. Trends Psychiatry Psychother..

[122] Vance A., Ferrin M., Winther J., Gomez R. (2013). Examination of Spatial Working Memory Performance in Children and Adolescents with Attention Deficit Hyperactivity Disorder, Combined Type (ADHD-CT) and Anxiety. J. Abnorm. Child Psychol..

[123] Vasa R.A., Roberson-Nay R., Klein R.G., Mannuzza S., Moulton J.L., Guardino M., Merikangas A., Carlino A.R., Pine D.S. (2007). Memory Deficits in Children with and at Risk for Anxiety Disorders. Depress. Anxiety.

[124] Klorman R., Hazel-Fernandez L.A., Shaywitz S.E., Fletcher J.M., Marchione K.E., Holahan J.M., Stuebing K.K., Shaywitz B.A. (1999). Executive Functioning Deficits in Attention-Deficit/Hyperactivity Disorder Are Independent of Oppositional Defiant or Reading Disorder. J. Am. Acad. Child Adolesc. Psychiatry.

[125] Dickerson Mayes S., Calhoun S.L., Crowell E.W. (1998). WISC-III Freedom from Distractibility as a Measure of Attention in Children with and without Attention Deficit Hyperactivity Disorder. J. Atten. Disord..

[126] Geurts H., Verte S., Oosterlaan J., Roeyers H., Sergeant J. (2005). ADHD Subtypes: Do They Differ in Their Executive Functioning Profile?. Arch. Clin. Neuropsychol..

[127] Mayes S.D., Calhoun S.L., Chase G.A., Mink D.M., Stagg R.E. (2009). ADHD Subtypes and Co-Occurring Anxiety, Depression, and Oppositional-Defiant Disorder: Differences in Gordon Diagnostic System and Wechsler Working Memory and Processing Speed Index Scores. J. Atten. Disord..

[128] Sarkis S.M., Sarkis E.H., Marshall D., Archer J. (2005). Self-Regulation and Inhibition in Comorbid ADHD Children: An Evaluation of Executive Functions. J. Atten. Disord..

[129] Schachar R., Tannock R. (1995). Test of Four Hypotheses for the Comorbidity of Attention-Deficit Hyperactivity Disorder and Conduct Disorder. J. Am. Acad. Child Adolesc. Psychiatry.

[130] Seidman L.J., Biederman J., Faraone S.V., Milberger S., Norman D., Seiverd K., Benedict K., Guite J., Mick E., Kiely K. (1995). Effects of Family History and Comorbidity on the Neuropsychological Performance of Children with ADHD: Preliminary Findings. J. Am. Acad. Child Adolesc. Psychiatry.

[131] Van Der Meer D., Hoekstra P.J., Van Rooij D., Winkler A.M., Van Ewijk H., Heslenfeld D.J., Oosterlaan J., Faraone S.V., Franke B., Buitelaar J.K. (2018). Anxiety Modulates the Relation between Attention-Deficit/Hyperactivity Disorder Severity and Working Memory-Related Brain Activity. World J. Biol. Psychiatry.

