# Supplementary material for: ADHD Children Take More Time to Inhibit Automatic Responses: A Comparison with Anxiety Disorders Using NEPSY-II
Source: Children (Basel). 2025 Jun 18;12(6):798. doi: 10.3390/children12060798 (PMC12191324; doi:10.3390/children12060798)
Supplement: Supplementary file 1 [file children-12-00798-s001.zip › children-3667951-supplementary.pdf]

**Supplementary Materials**

**Table S1:** Between-group differences in cognitive function measured through WISC-IV.

| *WISC-IV Index* | *Groups* | *M* | *SD* | *p Value* |
|---|---|---|---|---|
| *IQ* | *ADHD* | 106 | 10.2 | *ADHD vs. AD:* 0.931 |
| | *AD* | 104 | 14.6 | *ADHD vs. ADHD+AD:* 0.536 |
| | *ADHD+AD* | 100 | 7.59 | *AD vs. ADHD+AD:* 0.877 |

**Table S2:** Between-group differences in Naming (NEPSY-II Inhibition subtest, condition IN-A).

| *Naming (IN-A)* | *Groups* | *M* | *SD* | *p Value* |
|---|---|---|---|---|
| *Time scalar score* | *ADHD* | 7.95 | 3.37 | *ADHD vs. AD:* 0.669 |
| | *AD* | 9.07 | 3.01 | *ADHD vs. ADHD+AD:* 0.981 |
| | *ADHD+AD* | 8.36 | 2.25 | *AD vs. ADHD+AD:* 0.930 |
| *Combined scalar score* | *ADHD* | 8.91 | 3.04 | *ADHD vs. Anxiety:* 0.547 |
| | *AD* | 10.1 | 3.46 | *ADHD vs. ADHD+AD:* 0.968 |
| | *ADHD+AD* | 8.18 | 2.14 | *AD vs. ADHD+AD:* 0.410 |

**Table S3:** Between-group differences in Switching (NEPSY-II Inhibition subtest, condition IN-C).

| *Switching (IN-C)* | *Groups* | *M* | *SD* | *p Value* |
|---|---|---|---|---|
| *Time scalar score* | *ADHD* | 8.68 | 3.24 | *ADHD vs. AD:* 0.543 |
| | *AD* | 10.1 | 3.46 | *ADHD vs. ADHD+AD:* 0.911 |
| | *ADHD+AD* | 8.64 | 3.07 | *AD vs. ADHD+AD:* 0.955 |
| *Combined scalar score* | *ADHD* | 8.95 | 3.03 | *ADHD vs. AD:* 0.919 |
| | *AD* | 9.67 | 3.20 | *ADHD vs. ADHD+AD:* 0.963 |
| | *ADHD+AD* | 8.36 | 3.26 | *AD vs. ADHD+AD:* 0.756 |

**Table S4:** Between-group differences in Visual Attention (NEPSY-II).

| *Visual Attention* | *Groups* | *M* | *SD* | *p Value* |
|---|---|---|---|---|
| *Visual Targets* | *ADHD* | 22.1 | 7.45 | *ADHD vs. AD:* 0.727 |
| | *AD* | 19.8 | 6.00 | *ADHD vs. ADHD+AD:* 0.200 |
| | *ADHD+AD* | 17.2 | 6.54 | *AD vs. ADHD+AD:* 0.760 |
| *Visual Distractors* | *ADHD* | 5.73 | 9.33 | *ADHD vs. AD:* 0.916 |
| | *AD* | 5 | 8.08 | *ADHD vs. ADHD+AD:* 1.00 |
| | *ADHD+AD* | 3.91 | 3.24 | *AD vs. ADHD+AD:* 0.945 |
| *Visual Accuracy* | *ADHD* | 11.2 | 3.48 | *ADHD vs. AD:* 0.410 |
| | *AD* | 10 | 3.59 | *ADHD vs. ADHD+AD:* 0.307 |
| | *ADHD+AD* | 9.64 | 2.66 | *AD vs. ADHD+AD:* 0.991 |

**Table S5:** Between-group differences in Auditory Attention (NEPSY-II).

| *Auditory Attention* | *Groups* | *M* | *SD* | *p Value* |
|---|---|---|---|---|
| *Word targets* | *ADHD* | 27.5 | 1.82 | *ADHD vs. AD:* 0.927 |
| | *AD* | 26.7 | 7.49 | *ADHD vs. ADHD+AD:* 0.917 |
| | *ADHD+AD* | 28.5 | 3.31 | *AD vs. ADHD+AD:* 0.685 |
| *Omission Errors* | *ADHD* | 2.55 | 1.82 | *ADHD vs. AD:* 0.927 |
| | *AD* | 3.27 | 7.49 | *ADHD vs. ADHD+AD:* 0.917 |
| | *ADHD+AD* | 1.55 | 1.04 | *AD vs. ADHD+AD:* 0.685 |
